# Prediction of setup times for an advanced upper limb functional electrical stimulation system

**DOI:** 10.1177/2055668318802561

**Published:** 2018-11-18

**Authors:** Christine Smith, Laurence Kenney, David Howard, Karen Waring, Minxgu Sun, Helen Luckie, Nicholas Hardiker, Sarah Cotterill

**Affiliations:** 1 Department of Allied Health Professions, Sheffield Hallam University, Sheffield, UK; 2 School of Health Sciences, University of Salford, Salford, UK; 3 School of Computing, Science and Engineering, University of Salford, Salford, UK; 4 School of Nursing, Midwifery, Social Work & Social Sciences, University of Salford, Salford, UK; 5 Research & Development Department, Salford Royal NHS Foundation Trust, Salford, UK

**Keywords:** Rehabilitation devices, stroke, physiotherapist, upper limb, graphical user interface

## Abstract

**Introduction:**

Rehabilitation devices take time to don, and longer or unpredictable setup time impacts on usage. This paper reports on the development of a model to predict setup time for upper limb functional electrical stimulation.

**Methods:**

Participants’ level of impairment (Fugl Meyer-Upper Extremity Scale), function (Action Research Arm Test) and mental status (Mini Mental Scale) were measured. Setup times for each stage of the setup process and total setup times were recorded. A predictive model of setup time was devised using upper limb impairment and task complexity.

**Results:**

Six participants with stroke were recruited, mean age 60 (±17) years and mean time since stroke 9.8 (±9.6) years. Mean Fugl Meyer-Upper Extremity score was 31.1 (±6), Action Research Arm Test 10.4 (±7.9) and Mini Mental Scale 26.1 (±2.7). Linear regression analysis showed that upper limb impairment and task complexity most effectively predicted setup time (51% as compared with 39%) (F(2,21) = 12.782, adjusted *R^2^* = 0.506; *p* < .05).

**Conclusions:**

A model to predict setup time based on upper limb impairment and task complexity accounted for 51% of the variation in setup time. Further studies are required to test the model in real-world settings and to identify other contributing factors.

## Introduction

Numerous studies have demonstrated the potential for functional electrical stimulation (FES) technology to support upper limb recovery following stroke.^1,2^ FES devices have the potential to free up valuable therapist time and allow patients to practise upper limb training protocols outside of formal therapy time and at their own pace. In order to align with principles known to drive functional recovery, notably the need to intensively practise a variety of challenging, functional tasks,^3,4^ and offer sufficient flexibility to accommodate a broad range of patients, FES devices are becoming increasingly sophisticated. These advanced FES devices have multiple channels, are usually controlled via sensors, provide some form of biofeedback and may incorporate electrode arrays^2,5^ Although largely unreported, an unintended consequence of this increase in complexity is likely to have been an increase in setup time of some of these devices. For instance, systems that include a brain interface require calibration of the interface, and some of the recent upper limb systems based on iterative learning control rely on person-specific dynamic model identification.^2,6,7^

There is a clear challenge in making such advanced systems flexible to patients’ needs whilst ensuring that they are quick and easy for therapist and patient use^8^ Ease of use is a major barrier to FES devices being used in the patients’ home. In spite of the importance of short setup times, there is a scarcity of studies that have examined setup time for any form of rehabilitation device^9–11^ Even those that have reported setup time tend to rely on self-reports and do not clearly define setup time (when timing commenced and finished).^12,13^ The authors could not identify any papers reporting on setup time for upper limb rehabilitation devices.

Although it is clear that setup time should be as short as possible, one issue that has not been addressed in the literature is the need for setup time to be predictable and tools for this purpose do not appear to exist. Predictability of setup time is important from a clinical perspective, as sessions with patients are usually time-limited. A lengthy setup time or one that is unpredictable and variable may have consequences for technology adoption.

**Figure 1.**
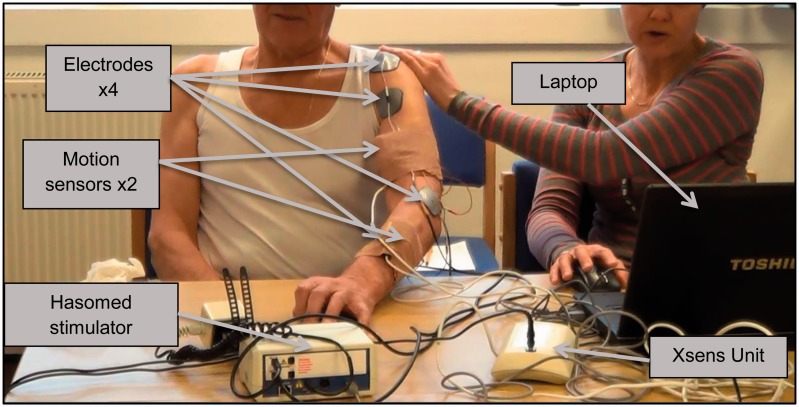
FESRT system comprising laptop, Xsens MTx Unit with two motion sensors, surface electrodes X4, Rehastim™ Hasomed stimulator.

In this paper, we present a model for the prediction of setup time for a new upper limb FES system, referred to as the FES Rehab Tool or FESRT (Figure 1). The FESRT is a clinic-based system designed to use electrical stimulation of weak or paralyzed muscles to support a range of people with upper limb impairments following stroke to practise a variety of functional activities they would be unable to perform unaided. The system is designed to be used under therapist supervision. The hardware consists of an eight-channel stimulator (RehaStim™ Hasomed); however, only four channels were utilised for our study, two body-worn inertial measurement units (Xsens MTx) and a laptop computer. The laptop runs purpose-designed software, written in Matlab-Simulink environment, for setting up and running state machine (sequential) controllers, specific to the activity and participant’s impairments. The state machine controller considers a particular functional activity as a sequence of movement phases, each of which is associated with stimulation to user-defined muscles at specified levels of intensity. Progress through the movement phases is governed by user-defined rules. These rules may use, as their input(s), angle data from the body-worn sensors, a button press using the laptop keyboard and/or time since entering the phase.^14^ To manage the setup and running of the FES controller, the user is provided with a graphical user interface (GUI). Threshold values for each muscle are established early in the setup process, leaving the therapist to define pulse width target and ramp time for each stimulated muscle in each phase. It is the prediction of the time taken to use this GUI to set up user-defined, FES-supported activities for particular patients which is the focus of this paper.

## Setup time model development

We use the word ‘model’ to describe the mathematical relationship between upper limb impairment and task complexity. In order to meet the requirements of our setup time model, a measure of task complexity was needed to fulfil the following characteristics:

- Be independent of impairment level, as this was represented in the other part of the model;
- Characterise functional movement for the upper limb, either using measures of joint or muscle activity, based on the assumption that the more changes of muscle or joint activity there are within a given task, the more complex the task is;
- Be applicable to ‘real-world’ functional tasks.

**Figure 2.**
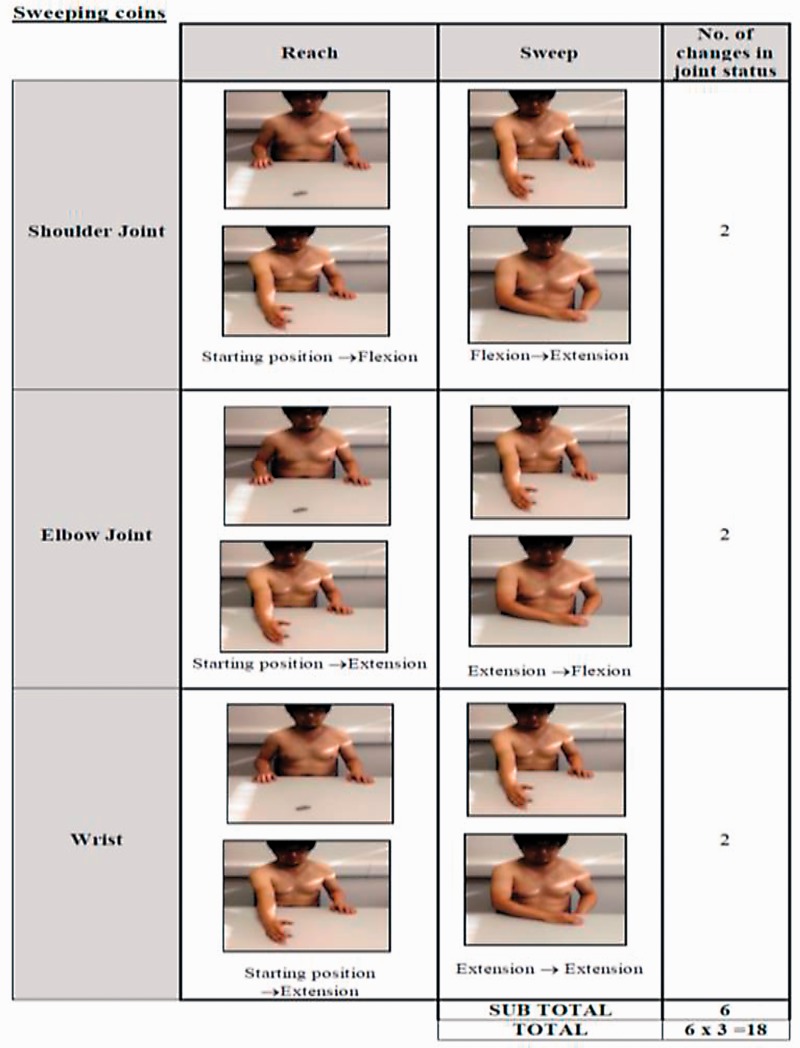
An example of the task, sweeping coins into contralateral hand. The columns that are entitled ‘Reach’ and ‘Sweep’ refer to the movement phase. Each picture shows the start and end point of the upper limb relative to each movement phase and anatomical joint.

No suitable model of task complexity was identified in the literature, and therefore a basic model was developed based on descriptions of joint movements that could be both directly observed and easily interpreted. The task complexity method focused on the movements of the major joints in the upper limb, shoulder, elbow, radio-ulnar joint and wrist, all of which could be controlled using FES. The model considered a task to consist of a number of phases. Within each phase, each of the four joints was considered to be in one of three conditions: (1) at rest; (2) moving in a single direction, e.g. flexion, extension, pronation and supination *or* (3) held in a static position, actively working to overcome any external forces. In order to illustrate how the task complexity calculation was arrived at, an example of a ‘sweeping coins’ task is provided in Figure 2. For a given task, the number of times a change in status occurred at each joint during each phase was recorded and the sum calculated. This number was then multiplied by the number of joints involved in the whole functional task, as a weighting factor. This took into account that tasks that involved co-ordinated movement at multiple joints were likely to be more complex than the sum of the complexity of individual joint movements (i.e. a movement involving coordination of two joints is likely more than twice as complex as a movement involving a single joint). This figure (i.e. sum of changes in joint status, multiplied by number of joints involved in the task) provided the task complexity score for a specific task.

To allow development of the tool to predict setup time, a suitable library of tasks needed to be identified that would be representative of those that might be used in a therapy session. The library of tasks took into account the importance of specificity of training,^15^ bilateral training due to the many tasks in everyday life that involve bilateral activity^16^ and the real-world relevance of objects in the tasks. Finally, we used results from a previous study^17^ which provided examples of functional tasks that are important to People with Stroke (PwS) and that they have difficulty in achieving.^18^

**Figure 3.**
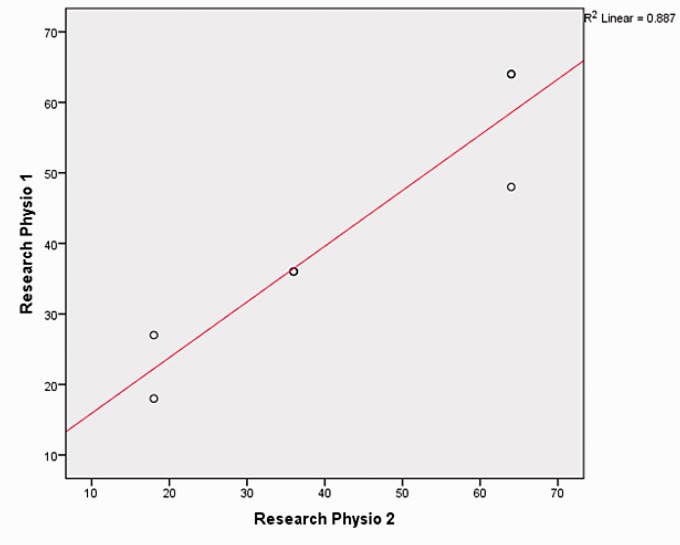
Scatterplot of task complexity scores for Research Physiotherapist 1 (author) and Research Physiotherapist 2. Line of the best fit included.

As the method for devising task complexity was study specific, it was important to ensure there was some robustness to this approach. A second senior research physiotherapist (RP) (RP2) was provided with the definition for calculating task complexity and asked to independently calculate the task complexity scores for the library of tasks. Based on each therapist’s individual scores, the set of tasks was ranked, placing the least complex task first and the most complex task last. Results were compared by plotting the results of RP1 against RP2 (Figure 3), including a line of best fit.

There was a high level of agreement across the RPs. The task complexity totals were the same for five of the seven tasks, and the ranking of task complexity was the same for all tasks. Of the two tasks where the scores differed, in each case, a movement phase had been omitted. After further discussion, it was agreed to include the additional movement phases (Table 1).

**Table 1.** Revised agreed scores in rank order (lowest to highest).

Revised agreed scores and ranking	
Task	Total score
Sweeping coins	18
Pushing up from chair	27
Place block on shelf	36
Picking up tray	36
Answering phone	64
Pouring from bottle	64
Opening door	64

## Methodology for testing the model

Ethical approval was gained from the Greater Manchester NHS Ethics committee (10/H1005/26) and the University of Salford Ethics Committees (REP10/146). Written informed consent for patient information and images to be published was provided by the patients. Seven chronic stroke participants who met the inclusion and exclusion criteria (Table 2) were purposively sampled from a database of volunteers. PwS who were deemed to represent the type of PwS most likely to benefit from an advanced FES system such as ours were selected. An information sheet was provided outlining the details of the study. Informed consent was gained on the first visit to the lab. Each participant was asked to visit the laboratory on up to six occasions.

**Table 2.** Lab-based testing: stroke participant inclusion and exclusion criteria.

Inclusion criteria
• A single stroke
• At least six months post stroke
• Medically stable
• Sufficient cognitive ability to understand the experimental protocols
• Over 18 years of age
• Adequate motor response to surface stimulation and able to tolerate sensation
• Reduced arm function on their hemiplegic side
Exclusion criteria
• Premorbid orthopaedic, neurologic or other medical condition including poorly controlled epilepsy, which would affect the response to electrical stimulation
• Cardiac demand pacemaker or other active medical implant/device that may be affected by FES
• Fixed contractures of elbow, wrist or fingers
• Pain due to shoulder subluxation

FES: functional electrical stimulation.

During the first visit, once informed consent had been provided, clinical data were gathered to characterise the participant. Their level of impairment was measured using Fugl-Meyer Upper Extremity (UE) Assessment, (FMA-UE).^19^ Other measures were also taken to characterise the participants as follows: Action Research Arm Test^20^ and Mini-Mental State.^21^ To remove one (external) source of variability in setup time, throughout testing, the same physiotherapist – who was trained to use the system and who specialised in stroke – carried out all the clinical measures and acted as the operator when setting up the FES device.

At subsequent visits, the same physiotherapist used the GUI to set up the FESRT for each of the tasks in the library, taking into account the participants’ level of capability. Where a task was either too easy (able to be completed without the use of FES) or too difficult (unable to be complete even with the assistance of FES), they were omitted. Where this situation arose, this information was recorded. Where possible, participants progressed through the tasks from simplest to most complex, in accordance with the task complexity ranking. This allowed participants to build confidence by successfully achieving some of the simpler tasks before being asked to attempt more complex tasks.

A usability data collection form was used to record time taken to set up each stage of the FES device and to record relevant usability observations during the setup process, for use in subsequent final refinement of the GUI. The timing of the setup process only began once all of the hardware was laid out and both the physiotherapist and the participant were ready to commence. Setting up the Hasomed FES Rehastim, the Xsens and loading the GUI (at this stage in the development, the software was loaded through Matlab commands) was carried out by an independent researcher who had written the code. This ensured that the FES system was set up consistently across all of the lab-based testing.

Times were captured using a stopwatch and were recorded from when the operator commenced stage 1 of the setup process. In order to test the lab-based protocol and the reliability of the software on stroke participants, the first participant (participant 0) was used as a pilot. The data from this participant were therefore not included in the results.

## Data analysis

We report the baseline characteristics of all the participants. The individual relationships between setup time and both task complexity and level of impairment (FM-UE) were explored using Pearson’s correlation and scatterplots. Scatterplots were conducted to visually establish the nature of any relationship between participants’ upper limb impairment scores and setup times for the FESRT. We used multiple linear regression to develop a model of the combined effect of impairment and task complexity on setup time, adding each variable in turn to establish whether adding both would improve the level of prediction. We report the overall fit of the model to predict setup time and the relative contribution of each of the independent variables. All analyses were done using SPSS V20.0. Statistical significance was set at *p* < .05, two tailed.

## Results

### Participant characteristics

All participants were classified as being in the chronic stage post stroke. Participants were graded using the Fugl-Meyer UE Scale as mild (50–65), moderate (30–49) or severe (below 30) according to the criteria used by Michelson et al.^22^ Four participants were therefore categorised as severely impaired, whilst two were moderate. Participants generally had a low level of functional ability. One participant had expressive language difficulties as a result of the stroke. All other participants had no communication or language deficits (Table 3).

**Table 3.** Participant characteristics: impairment, function and Mini Mental scores for the lab-based testing.

Participant no.	Time since CVA (years)	Age (years)	Affected side	Hand dominance	Gender	FM UE/66	ARAT/57	Mini Mental/30
1	28	59	R	R	M	18	4	24
2	3	80	L	R	M	29	10	26
3	5	41	R	R	M	29	8	23
4	3	79	L	R	M	28	6	25
5	13	42	R	R	F	37	NK	30
6	7	59	L	R	M	38	24	29
Mean	9.8 (±9.6)	60 (±17)				31.1 (±6)	10.4 (±7.9)	26.1 (±2.7)

NK: not known; FM UE: Fugl-Meyer Upper Extremity Scale; ARAT: Action Research Arm Test; CVA: Cerebrovascular Accident.

### Setup times

Setup times were recorded for each stage (stages 1–4) of the setup process together with the overall setup time for each of the seven functional tasks. Table 4 displays the overall initial setup times (min) for each participant, per completed task. A key for the functional task code is also provided.

**Table 4.** Impairment level and setup times per participant and functional task.

Participant Number	Age (years)	FM-UE	Task SC (18)	Task PC (27)	Task BS (36)	Task PT (36)	Task OD (64)	Task PB (64)	Task AP (64)
1	59	18	28.51	38.93					50.71
2	80	29	29.33		37.51	39.36	23.88	49.85	41.73
3	41	29	20.98		49.80	33.96			
4	79	28	23.31	23.90		27.30			37.56
5	42	37	14.50			16.08		17.15	22.38
6	59	38	19.71			16.32		18.11	24.15
Mean	60 (±17)	29.8 (±7.2)	22.7 (±5.6)	31.4 (±10.6)	43.6 (±8.6)	26.6 (±10.4)	N/A	28.3 (±18.6)	35.3 (±11.9)

Note: Task complexity scores are shown in brackets. Grey shaded regions represent task not attempted. SC: sweeping coins; PC: pushing up from chair; BS: place block on shelf; PT: picking up tray; OD: opening door; PB: pouring from bottle; AP: answering phone.

There was a general trend for setup time to increase with task complexity and a general trend for the setup time to be longer with patients with greater levels of impairment.

### Relationship between task complexity and setup times and the level of patients’ upper limb impairment and setup times

There was a weak positive correlation (0.23) between task complexity and setup time which was not statistically significant. However, there was a large negative relationship between the participants’ level of impairment and setup time, with a Pearson correlation coefficient of −0.643 (*p* < .05).

### Modelling setup time

A linear regression model (Table 5) showed that each additional increase (improvement) in upper limb impairment score reduces setup time by an average of 1.28 min, after taking into account the effect of task complexity. Similarly, each increase in task complexity score increases setup time by an average of 0.22 min after taking into account the effect of impairment score, and both were statistically significant (*p* < .05)

**Table 5.** Summary of multiple regression analysis and the effect of task complexity and upper limb impairment on setup time.

	Model	
Variable	Coef.	SE
Intercept	59.04	8.50
Impairment	−1.28*	0.26
Task complexity	0.22*	0.08
Adjusted *R*^2^	0.51*	

Coef.: unstandardised regression coefficient.

* *p* < .05.

As derived from the regression analysis, the equation to predict setup time was

$$\begin{array}{c} \text{Predictedsetuptime}=59.042-(1.28\times \text{impairment})\\ +(0.22\times \text{taskcomplexity}). \end{array}$$

### How well did the proposed model fit?

When corrected for any positive bias, the adjusted *R^2^* of Model 2 was 0.51 (51%), indicative of a medium to large combined effect^23^ of impairment and task complexity on setup time.

## Discussion

### Model development

#### Factors likely to influence setup time

**Figure 4.**
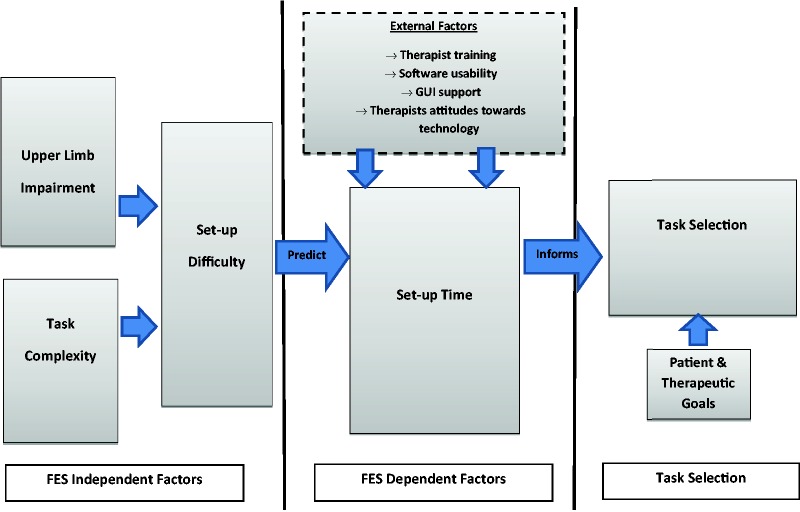
The inter-relationship between upper limb impairment, task complexity and additional factors when predicting setup time and task selection.

We proposed that setup time was likely to be influenced in the first instance by two factors (Figure 4). First, the level of *upper limb impairment*. For individuals with no impairment and hence requiring no FES support, setup time should be zero. Conversely, an individual with a high level of impairment, attempting the same task, would require a high degree of assistance from the system. It was reasonable therefore to propose that for a given task, the number of channels of stimulation, and hence the associated time needed to place electrodes and find appropriate stimulation levels, would be positively related to the patients’ level of impairment. The second factor was the *complexity of the task* to be practised. We also proposed that a simple task, involving a small number of movement phases, should take less time to set up than a complex task involving more movement phases, as setting up of each rule between movement phases has an associated time cost. It was postulated that a model based on *impairment* and *task complexity* may allow for prediction of setup time.

From the regression analysis, the participants’ level of upper limb impairment, as measured by the Fugl-Meyer UE Scale, appeared to have the greatest influence within the proposed model on the prediction of setup time for the UL FESRT. Generally speaking, the more impaired the participant, the greater the overhead in terms of setup. Within the range of tasks selected, task complexity appeared to have less influence on setup time. This finding was consistent with the author’s observations.

#### Internal factors affecting setup time

- Upper limb impairment

Although the proposed model and findings from the lab-based testing appear promising, it is important to recognise that the model only predicted 51% of the variance in setup time and hence needs further refinement. Other patient characteristics such as presence of spasticity, cognitive involvement or communication deficits can potentially impact the setup times. Although one of the participants recruited for the lab testing had expressive language difficulties, this participant was well known to the testing team, resulting in minimal increase in setup time. Introducing other variables into the model at this stage of the development process was not possible, as this would have required additional testing to gather more data. In addition, although the Fugl-Meyer UE Scale was felt to be a reliable and valid measure of impairment, other measures of impairment may offer a more sensitive measure of impairment level. The model only applies to people with some form of neurological impairment. Clearly, the model is invalid for people with no impairment.

- Task complexity

Although task complexity also significantly contributed to the prediction of setup time, it contributed less than participants’ impairment scores. The method of calculating task complexity provided a useful starting point that allowed exploration of the relationship between task complexity and upper limb impairment and subsequently the effect of these variables on setup. In the current study, a pragmatic approach was adopted, that merely aimed to refine the scoring of the set of tasks, using two raters. However, as the method appears to have some merits, more formal reliability testing would be warranted. It is worthy of note that the proposed method is only applicable for the range of tasks included in the lab-based testing. It remains to be seen how well the method generalises to other functional tasks.

#### External factors affecting setup time

There are other factors that potentially influence setup time for FES devices outside of the lab (Figure 4). First, the effectiveness of training that therapists receive is critical to effective use and indeed adoption of rehabilitation devices. Hochstenbach-Waelen and Seelen^8^ highlighted the need for therapists to become familiar with technology by spending time at workshops and learn from peers whilst using the device. One way of mitigating against the impact of time away from patients in the clinical setting would be for rehabilitation technology to feature more prominently in therapists’ pre-registration education. Presently, there is only a small amount of time dedicated to rehabilitation technologies in the majority of pre- and post-qualification curricula. Second, the usability of the software and indeed its level of robustness have the potential to influence setup times.^24,25^ In the current study, usability factors such as the amount of support the GUI provided to the therapist was unchanged throughout testing. Finally, the model has only been developed for a single system (the FESRT). Further work would be needed to explore to what extent the two factors (impairment and task complexity) might influence setup time of other upper limb rehabilitation devices.

## Limitations and conclusions

### Limitations

The number of participants in the study was bounded by the resources available. However, there was a sufficient number to identify potential key influencers on setup time. The impairment profile of these participants was limited to participants categorised as either moderate or severely affected. This meant that it was not possible to ascertain if the model would have generalised to participants with only mild levels of impairment. In addition, these participants were all in the chronic stage of stroke and therefore at this point it was not possible to determine if the proposed model of calculating setup time would generalise to participants in the acute or sub-acute phases post stroke. Testing in the lab, in only a partially controlled environment, at times proved to be challenging when attempting to standardise the method for timing the setup process. However, every attempt was made to ensure any disruption to the timing of setup was excluded from the setup time calculations. Although the model to predict setup time for the FESRT is useful to therapists as part of the decision process when selecting which task to choose for participants, it cannot be generalised to other rehabilitation technologies.

### Conclusions

This is the first model that has attempted to predict setup time for a rehabilitation technology, namely, FES. The model, based on participants’ level of upper limb impairment combined with a task complexity score, predicted initial setup time for participants in the chronic stage post stroke. However, further testing needs to be carried out on participants in the acute and sub-acute stages post stroke and on participants with only a mild level of impairment. In addition, it remains to be seen if the model will apply when the FESRT is used in a real-world clinical environment. While these outstanding issues must await future studies, it is our hope that this seminal work will inform related research into rehabilitation technologies.
